# Simple two-step fabrication method of Bi_2_Te_3 _nanowires

**DOI:** 10.1186/1556-276X-6-277

**Published:** 2011-04-04

**Authors:** Joohoon Kang, Jin-Seo Noh, Wooyoung Lee

**Affiliations:** 1 Department of Materials Science and Engineering, Yonsei University, 262 Seongsanno, Seodaemun-gu, Seoul 120-749, Korea

## Abstract

Bismuth telluride (Bi_2_Te_3_) is an attractive material for both thermoelectric and topological insulator applications. Its performance is expected to be greatly improved when the material takes nanowire structures. However, it is very difficult to grow high-quality Bi_2_Te_3 _nanowires. In this study, a simple and reliable method for the growth of Bi_2_Te_3 _nanowires is reported, which uses post-sputtering and annealing in combination with the conventional method involving on-film formation of nanowires. Transmission electron microscopy study shows that Bi_2_Te_3 _nanowires grown by our technique are highly single-crystalline and oriented along [110] direction.

## Introduction

Low-dimensional nanostructures have received great attention due to their unique and unusual properties in many research fields related to nanoscience and nanotechnology [1]. One of the low-dimensional nanostructures, namely the one-dimensional (1D) nanowire, has a high aspect-ratio, making it suitable for future electronic and thermoelectric devices and new types of sensors [2,3]. In particular, it is believed that the classical size effect and quantum confinement effect in 1D nanowire play a crucial role in enhancing thermoelectric performance [1,4,5]. Bismuth telluride (Bi_2_Te_3_) is well known for its high thermoelectric figure-of-merit (*ZT *~ 1) in bulk. Moreover, its thermoelectric performance is expected to be remarkably improved for nanowire structures as a consequence of the high thermoelectric power (*S*^2^*σ*) and suppressed thermal conductivity (*κ*) in the low-dimensional structures [6,7]. More recently, Bi_2_Te_3 _has also been intensively investigated for the search of an efficient topological insulator since the observation of the quantum-spin-Hall-like phenomenon on the surface of a material even without the applied magnetic fields. Topological insulator materials show almost dissipationless surface conduction because of the high spin degeneracy caused by the spin--orbit coupling, although they behave like an insulator in bulk. Unlike the bulk Bi_2_Te_3_, the existence of the surface states in 1D Bi_2_Te_3 _nanowires has been predicted only by theory [8,9]. Since the theoretical expectation, numerous synthesis methods of Bi_2_Te_3 _nanowires have been developed over the past several years [10-16]. As part of such efforts, we have already reported the simple Bi_2_Te_3 _nanowire growth using a stress-induced method with no catalysts, starting materials, and templates, which is called the on-film formation of nanowires (OFF-ON) [17,18]. However, the one-step compound nanowire growth using this method is hard to establish the optimum conditions because diffusivity difference between multiple components often leads to nanowires grown with compositions different from a nominal stoichiometry in the thermal annealing step. In this article, a more reliable Bi_2_Te_3 _nanowire growth method is reported based on the OFF-ON process. Our approach is a two-step OFF-ON process. The first step involves pure Bi nanowire growth by the conventional OFF-ON method [17]. The second step is the *in situ *deposition of Bi_2_Te_3 _thin film onto a substrate including pure Bi nanowires, followed by thermal annealing. Bi_2_Te_3 _nanowires are synthesized through the inter-diffusion of constituent elements between the Bi nanowire core and the Bi_2_Te_3 _shell during this second step. Here, the reliability of this Bi_2_Te_3 _nanowire growth process and the quality of single-crystalline Bi_2_Te_3 _nanowires thus grown will be presented.

## Experiment

**Figure 1:**
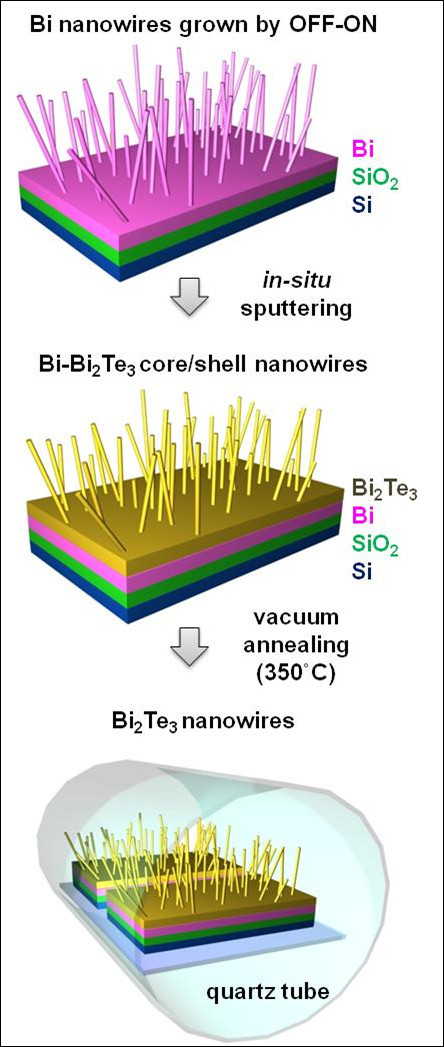
**Schematic representation of Bi_2_Te_3 _nanowire synthesis method**. Step 1: Bi nanowires are grown on the oxidized Si substrate by the OFF-ON method. Step 2: Bi_2_Te_3 _is deposited onto the substrate containing the Bi nanowires by *in situ *RF sputtering, which forms Bi-Bi_2_Te_3 _core/shell nanowires. Homogeneous Bi_2_Te_3 _nanowires are synthesized during the vacuum annealing at 350°C.

Figure 1 illustrates the schematics of Bi_2_Te_3 _nanowires synthesis process based on the OFF-ON method. To synthesize Bi_2_Te_3 _nanowires, Bi nanowires are grown by the OFF-ON method in the first step [17]. For Bi nanowire growth, a Bi thin film is first deposited onto a SiO_2_/Si substrate at a rate of 32.7 Å/s by radio frequency (RF) sputtering under a base pressure of 10^-7 ^Torr. Then, the Bi film on the SiO_2_/Si substrate is thermally annealed at 250°C for 10 h in an ultrahigh vacuum to grow Bi nanowires. Bi nanowires spontaneously grow to release the compressive stress acting on the Bi film, which is produced by the large thermal expansion coefficient difference between a Bi thin film (13.4 × 10^-6^/°C) and a SiO_2_/Si substrate ((0.5 × 10^-6^/°C)/(2.4 × 10^-6^/°C)) [17]. After the Bi nanowire growth is completed, a Bi_2_Te_3 _thin film is deposited onto the Bi nanowire-including SiO_2_/Si substrate using *in situ *RF sputtering under a base pressure of 10^-7 ^Torr. The samples then undergo vacuum annealing at 350°C for 10 h. During this second step, Bi_2_Te_3 _nanowires are synthesized, as the component atoms are inter-diffused between the Bi core nanowire and the Bi_2_Te_3 _surface layer. Moreover, the excess Bi atoms evaporate due to the high annealing temperature (350°C) well above the melting point of Bi (271.5°C), leaving behind stoichiometric Bi_2_Te_3 _nanowires. The probability of Te evaporation is expected to be low, since the annealing temperature (350°C) is significantly lower than the melting points of Te (449.5°C) and Bi_2_Te_3 _(585°C). The whole process is very simple, as schematically depicted in Figure 1. To characterize Bi_2_Te_3 _nanowires in detail, atomic structure, crystalline quality, and composition are analyzed using high-resolution transmission electron microscopy (HR-TEM).

## Results and discussion

**Figure 2:**
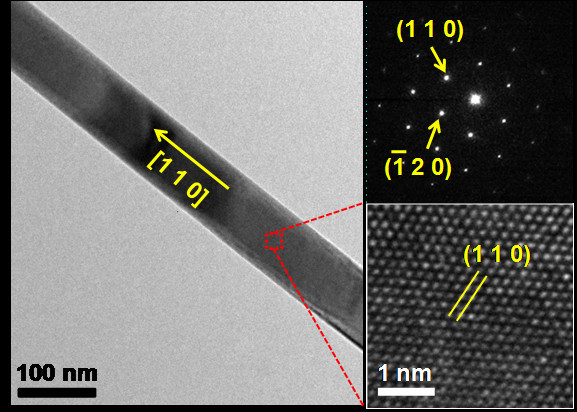
**A low-magnification TEM image shows an individual Bi_2_Te_3 _nanowire with a diameter of 78 nm**. A SAED pattern reveals that the Bi_2_Te_3 _nanowire is grown in [110] direction with high single-crystallinity. A high-resolution TEM image also indicates highly single-crystalline atomic arrangements without any defects.

TEM analyses of Bi_2_Te_3 _nanowires grown by the two-step process were performed. Bi_2_Te_3 _nanowires have a cylindrical shape, several tens of nanometers in diameter and several hundreds of micrometers in length. Figure 2 exhibits representative TEM images of a Bi_2_Te_3 _nanowire with a diameter of approximately 80 nm. From the selected area electron diffraction (SAED) pattern in the direction perpendicular to the longitudinal axis of the nanowire, it can be recognized that the Bi_2_Te_3 _nanowire is highly single-crystalline and its growth direction is [110]. A HR-TEM image confirms that the Bi_2_Te_3 _nanowire is oriented to [110] the direction with single-crystalline and defect-free atomic arrangements.

**Figure 3:**
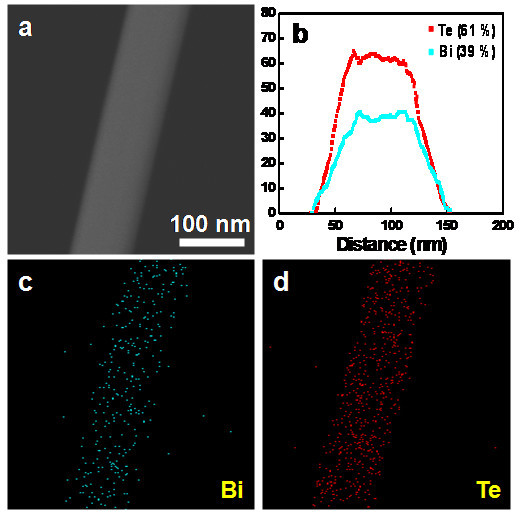
**Composition analysis of a Bi_2_Te_3 _nanowire**. **(a) **A HAADF image of the Bi_2_Te_3 _nanowire. **(b) **EDS line scan profiles showing the distributions of Bi (cyan, 39%) and Te (red, 61%) through the nanowire. **(c,d) **Elemental mapping images show the uniform distributions of Bi (cyan) and Te (red) along the nanowire.

To confirm the chemical composition of the Bi_2_Te_3 _nanowires, scanning TEM (STEM) and energy dispersive X-ray spectroscopy (EDS) were utilized. Figure 3a is a high-angle angular dark field (HAADF) STEM image of a Bi_2_Te_3 _nanowire with a diameter of 78 nm. The EDS line scan profiles show the uniform atomic distribution of Bi and Te elements through the whole nanowire, as displayed in Figure 3b. More importantly, the atomic ratios of Bi and Te are analyzed to be 39 ± 1 and 61 ± 1%, respectively. This reveals that the nanowire is composed of the thermodynamically stable, stoichiometric Bi_2_Te_3 _phase within the measurement error of STEM. The composition of Bi:Te = 2:3 is further confirmed by STEM elemental mappings across the same nanowire (see Figure 3c, d).

**Figure 4:**
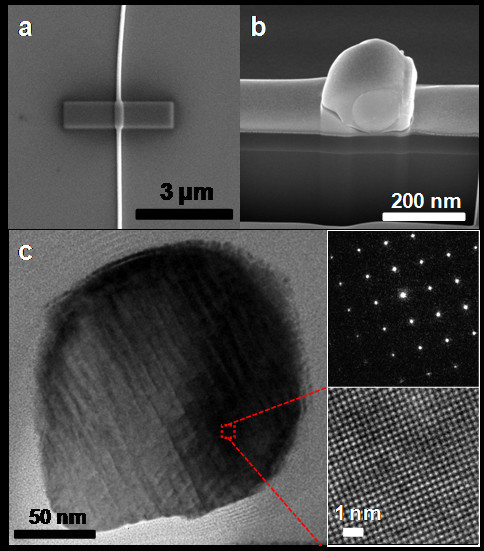
**A cross section of a Bi_2_Te_3 _nanowire**. **(a) **Pt is deposited locally to protect Bi_2_Te_3 _nanowire during the dual beam FIB process. **(b) **A SEM image shows the cross section of Bi_2_Te_3 _nanowire. **(c) **A low-magnification TEM image of the cross section of Bi_2_Te_3 _nanowire. There is no interface between the original Bi core and the Bi_2_Te_3 _shell after annealing. A SAED pattern and a HR-TEM image reveal that Bi_2_Te_3 _nanowire is highly single-crystalline across the nanowire.

Because our method for Bi_2_Te_3 _nanowires synthesis uses heterogeneous nanowire structures consisting of OFF-ON-grown Bi core and post-deposited Bi_2_Te_3 _shell, the homogeneity of final nanowires should be verified. The biggest concern may be a residual existence of an interface between the original core and the shell layers. To examine this possibility, cross-sectional TEM measurements of thin slices randomly taken from the nanowires were carried out. For the TEM sampling, dual-beam focused ion beam (FIB) was utilized based on the process depicted in Figure 4. Pt was deposited onto a Bi_2_Te_3 _nanowire to prevent any distortion during the dual-beam FIB processes (Figure 4a). Focused gallium (Ga) ion beam or electron beam generated from a fine nozzle makes it possible to deposit or etch a Pt film area selectively on the substrate. The Ga ion beam dissociates injected Pt-precursor molecules and removes the ligands from them on the selective area, resulting in local deposition of the Pt film. This is the well-known technique for TEM sampling [19]. Then, the Omni-probe of the dual-beam FIB tool took the etched TEM sample with a thickness of below 100 nm away from the SiO_2_/Si substrate. The final sample for TEM measurement is shown in Figure 4b. Figure 4c is the cross-sectional TEM image of a Bi_2_Te_3 _nanowire. From a HR-TEM image and SAED pattern of the part where a Bi core-Bi_2_Te_3 _shell interface was originally located, it is found that the synthesized Bi_2_Te_3 _nanowire has no interface inside and is crystalline across the cross section. These results indicate that the inter-diffusion of component atoms actively occurs between the Bi core and the Bi_2_Te_3 _shell during a 10-h annealing at the elevated temperature, with evaporation of excess Bi atoms at the nanowire surface.

## Conclusions

A simple and new synthesis method of quality single-crystalline Bi_2_Te_3 _nanowires combining the OFF-ON method with post-sputtering and annealing is demonstrated. In step one, Bi nanowires are grown by the conventional OFF-ON method. In step two, a Bi_2_Te_3 _thin film is *in situ *deposited onto the Bi nanowire-including substrate by RF sputtering, followed by the post-annealing at a high temperature well above the melting point of Bi. Bi_2_Te_3 _nanowires are synthesized during the high-temperature annealing by the atomic inter-diffusion between the Bi core and the Bi_2_Te_3 _shell. Indeed, our two-step growth method yielded homogeneous, stoichiometric Bi_2_Te_3 _nanowires with high single-crystallinity and no observable defects, which were hard to achieve using the conventional OFF-ON growth from a single compound source. These results are expected to facilitate the studies on high-efficiency thermoelectric devices and topological insulators taking advantage of Bi_2_Te_3 _nanowires.

## Abbreviations

EDS: energy dispersive X-ray spectroscopy; HAADF: high-angle angular dark field; HR-TEM: high-resolution transmission electron microscopy; OFF-ON: on-film formation of nanowires; RF: radio frequency; SAED: selected area electron diffraction; STEM: scanning TEM.

## Competing interests

The authors declare that they have no competing interests.

## Authors' contributions

J.K carried out this nanowire growth experiment and character analysis and drafted the manuscript. J-S.N participated in the design of the experiment and revised the manuscript. These whole experiment, analysis, and manuscript are totally directed by Prof. W.L. All authors read and approved the final manuscript.
